# Rare case of primary cutaneous adenoid cystic carcinoma of the abdomen: A case report

**DOI:** 10.1177/2050313X221086320

**Published:** 2022-03-30

**Authors:** Jeffrey Chivinski, Kevin Watters, Alexandra Mereniuk

**Affiliations:** 1 Department of Medicine, Division of Dermatology, Université de Montréal, Montreal, QC, Canada; 2 Department of Medicine, Division of Dermatology, Hôpital du Sacré-Coeur de Montréal, Montreal, QC, Canada; 3 Department of Pathology, McGill University Health Centre, Montreal, QC, Canada

**Keywords:** Dermatology, pathology, cancer

## Abstract

Adenoid cystic carcinoma is predominantly a tumor of the parotid glands and can sometimes be found in other glands. In most cases, skin location is usually a metastatic presentation and rarely a primary tumor. We describe the case of a 59-year-old female patient presenting with a 5-mm skin-colored nodule on the abdomen histologically compatible with a primary cutaneous adenoid cystic carcinoma. Extensive workup revealed no other primary source, nor evidence of metastatic disease; therefore, wide local excision was the preferred treatment given the low potential of recurrence. As this adnexal carcinoma is rare and its morphology non-specific clinically, we wanted to raise awareness of this entity and its management.

## Introduction

Formally described for the first time by Boggio^1^ in 1975, primary cutaneous adenoid cystic carcinoma (PCACC) is a very rare malignant skin appendageal tumor that typically manifests on the head and neck areas. Occasionally metastasis can arise, thus meticulous investigation is required. The scientific literature reports only a few case series^2^ and its clinical presentation may be non-specific, emphasizing the importance of immunochemistry to distinguish this entity from other differentials. We present the case of a 59-year-old female with an abdominal PCACC as these tumors rarely develop in this location and are often misinterpreted as other carcinomas.

## Case report

**Figure 1.**
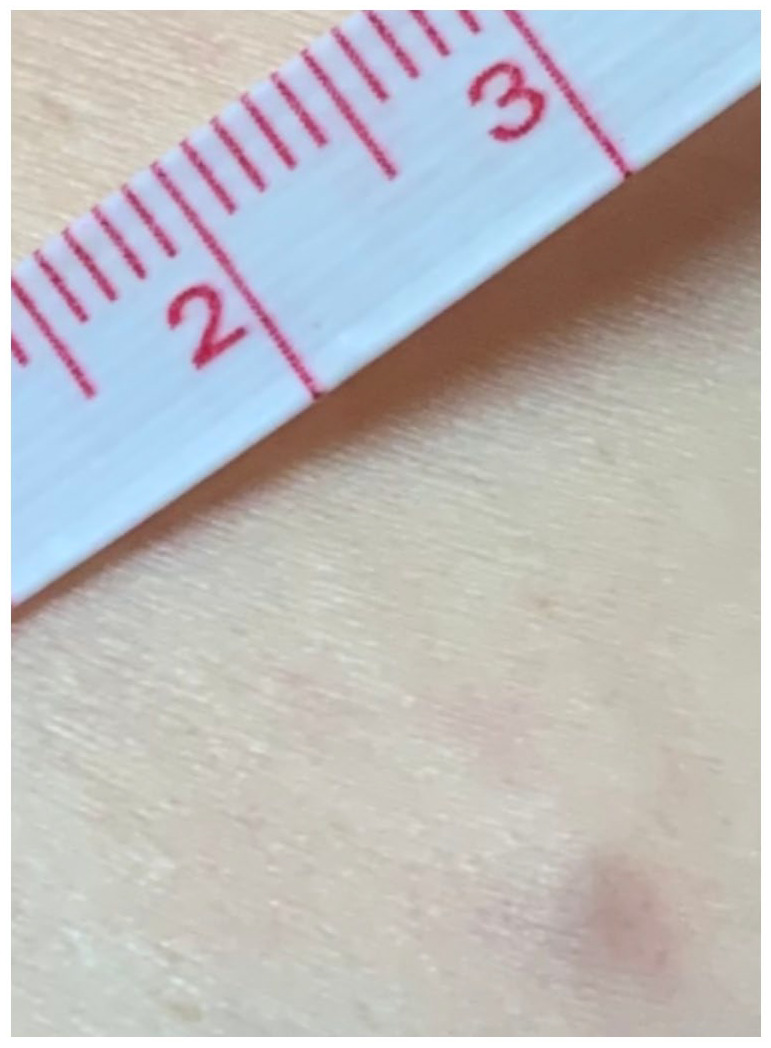
Non-specific skin-color papule on the abdomen.

A 59-year-old female patient presented with a tender nodule on her left abdomen. It had been noted by the patient 7 months prior, without further major change or expansion since. Past medical history was unremarkable. Physical examination showed a well-circumscribed skin-colored nodule located on the left lower abdominal quadrant. It displayed no epidermal changes and measured 5 mm (Figure 1). A complete skin exam did not reveal other lesions, palpable lymph nodes or organomegaly.

**Figure 2.**
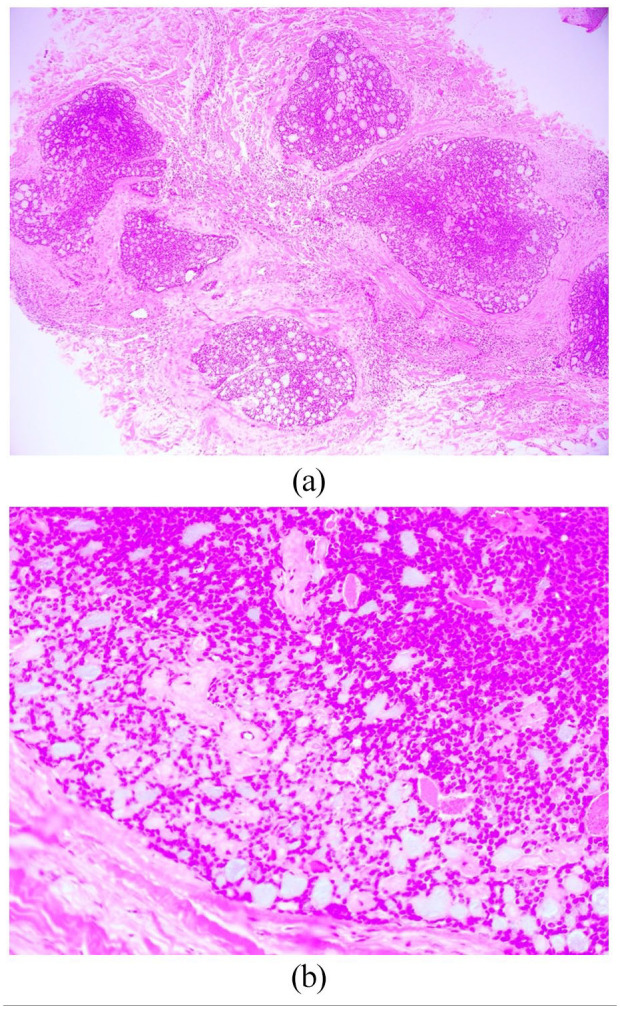
(a) H&E low-power (×4): Lobular mainly cribriform tumor of small basaloid cells with mucinous pseudocysts. (b) H&E high-power (×40): Lobular mainly cribriform tumor of small basaloid cells with mucinous pseudocysts.

**Figure 3.**
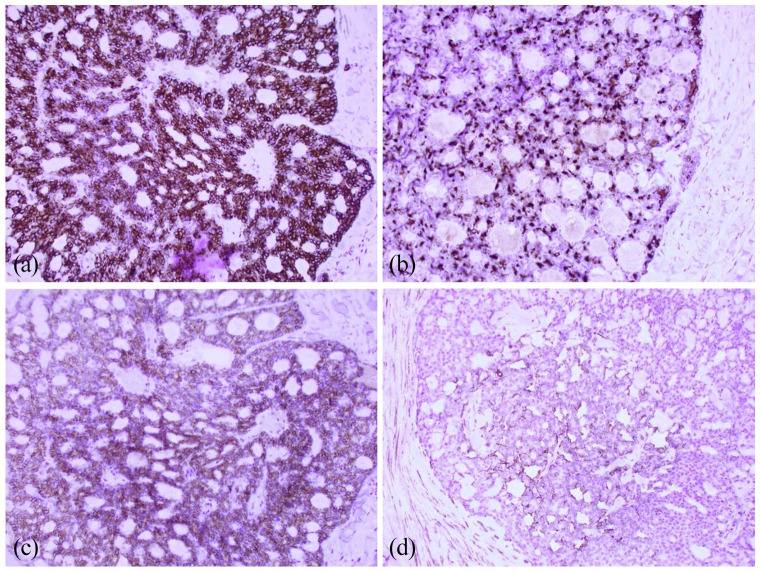
Immunohistochemistry: (a) CK7; (b) EMA; (c) CD117; (d) HHF35.

An excisional biopsy was performed and showed a mid to deep dermal lobular basaloid tumor with a prominent adenoid pattern (Figure 2). Mucin secretion was present and immunohistochemistry revealed significant positivity in tumor cells for CD117, CK7, AE1/AE3A, EMA and HHF35 with focal positivity of S100 (Figure 3). There was no vascular or perineural invasion.

Findings were compatible with an adenoid cystic carcinoma (ACC). As ACC is most often a primary tumor of the salivary glands, the patient was referred to otorhinolaryngology to exclude a primary tumor in this location. Nasopharyngoscopy did not reveal suspicious lesions, and computed tomography (CT) scan showed normal parotid and salivary glands. In order to thoroughly rule out other potential sites of primary involvement, the patient was also referred to gynecology and oncology. Mammogram and pap smear were negative. Thoraco-abdomino-pelvic CT scan did not reveal any suspicious lesions. Due to a low potential of metastasis, the oncology team judged it was unnecessary to perform a positron emission tomography scan (PET scan). Re-excision with wide margins was considered to be sufficient as treatment, without adjuvant radiotherapy. To this day, no recurrence of the tumor has been noted.

## Discussion

ACC is a slow-growing tumor predominantly involving the salivary glands, and to a minor extent breasts, lacrimal glands, bronchi or the uterine cavity and cervix.^3^ PCAAC is a rare variant of this adnexal carcinoma of apocrine lineage and may be impossible to distinguish from a metastatic ACC, underlining the importance of searching for a primary lesion elsewhere. It presents as a solitary skin-colored firm nodule with an indolent progressive course. Ever since it was first described by Boggio in 1975, the scientific literature has reported few case series and lately one large cohort analysis in the United States of 451 cases over 40 years.^2,4,5^ The majority of tumors occurred in the head and neck in elderly and middle-aged patients.^2,4^ Less often, it can be found on the trunk^6–8^ or extremities.^9^ Unlike ACC that can metastasize to lungs, bones and soft tissues, PCACC rarely metastasizes^10^ but can display local perineural involvement with a tendency to reoccur locally.^11^ As PCACC might resemble micronodular basal cell carcinoma (BCC) on histopathology, distinctive features help to rule out other differentials.

Classical PCACC histopathology comprises basaloid cells in the dermis and hypodermis with no epidermal connection. These basaloid cells can be arranged in cribriform structures (net-like bridges between ductal spaces), tubules, cords and solid areas with rare myxoid stroma. Batsakis and Luna^12^ described a tumor grading system for ACC depending on these specific patterns present on histology. The tumor cells generally follow two identifiable differentiation patterns: myoepithelial and ductal. Immunochemistry markers are crucial to identify the two cell populations and provide additional diagnostic support. EMA, CK7 and CD117 are expressed in the ductal structures, while focal positivity for S100 protein and SMA (HHF35) (smooth muscle actin) highlight the myoepithelial cells.^13,14^ Markers also permit the distinction between similar histopathological presentations. Indeed, adenoid basal cell carcinomas present cribriform structures with basaloid cells, but will usually lack positivity for S100, CK7, CD117 and EMA.^2^ Other differential diagnoses include primary cutaneous cribriform carcinoma, micronodular BCC, spiradenoma, mucinous carcinoma and other adnexal carcinomas.

Treatment of PCACC is essentially surgical, with most cases being treated by wide excision of the tumor. In the presence of larger tumors or nodal spreading, radiation may be used. Rarely chemotherapy is utilized in metastatic disease. Because of frequent perineural involvement, local recurrences are common and reported in up to 25% of cases.^2^ The 5-year survival rate is estimated at 96.1%, with an inferior prognostic for trunk involvement (75.6%).^4^

Our article presents a rare case of uncomplicated abdominal PCACC. Although truncal location of this tumor is rare, histopathology and immunochemistry showed a classic presentation of basaloid cell pattern with ductal and myoepithelial differentiation. Given the rarity of PCACC, most authors recommend excluding cutaneous metastatic disease from a primary tumor of more classic localization. Our patient had an extensive workup confirming primary cutaneous disease. Given the absence of perivascular and perineural involvement, the risk of recurrence was deemed very low after wide excision of the tumor. Clinical follow-up is suggested, nonetheless. As this adnexal carcinoma is rare and its morphology non-specific clinically, we wanted to raise awareness of this entity and its management.
